# Supplementary material for: A loss-of-function mutation in RORB disrupts saltatorial locomotion in rabbits
Source: PLoS Genet. 2021 Mar 25;17(3):e1009429. doi: 10.1371/journal.pgen.1009429 (PMC7993613; doi:10.1371/journal.pgen.1009429)
Supplement: S1 Fig — Gene expression was measured through quantitative RT-qPCR of two amplicons in three rabbit individuals, one per genotype: wild-type (+/+), heterozygote (+/sam) and sauteur (sam/sam). The y-axes indicate a relative measure of expression of each amplicon controlled for the expression of a housekeeping gene (GAPDH). Main bars indicate average relative expression, and error bars indicate the minimum and maximum values of three technical replicates for each tissue/individual. (PDF) [file pgen.1009429.s001.pdf]

S1 Fig

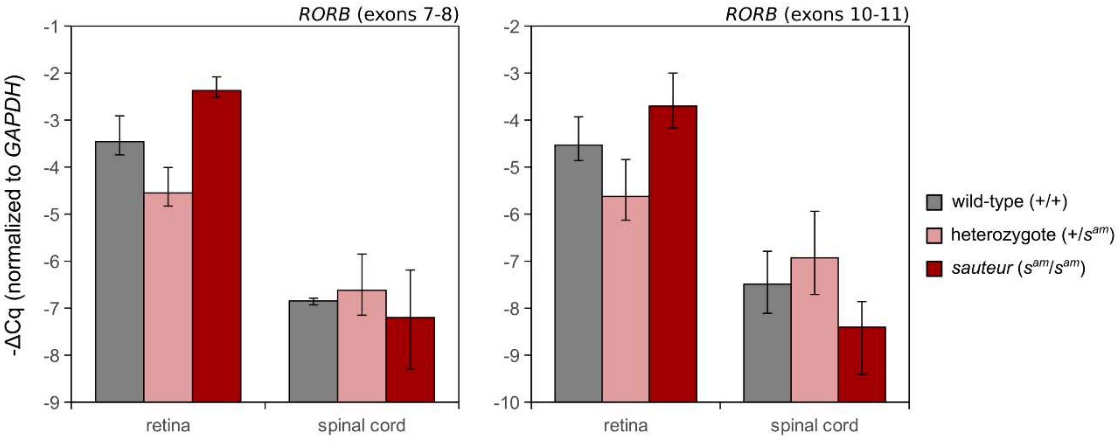

S1 Fig. Gene expression levels of RORB in the retina and spinal cord. Gene expression was measured through quantitative RT-qPCR of two amplicons in three rabbit individuals, one per genotype: wild-type (+/+), heterozygote (+/s^{am}) and sauteur (s^{am}/s^{am}). The *y*-axes indicate a relative measure of expression of each amplicon controlled for the expression of a housekeeping gene (*GAPDH*). Main bars indicate average relative expression, and error bars indicate the minimum and maximum values of three technical replicates for each tissue/individual.
