# Supplementary material for: A loss-of-function mutation in RORB disrupts saltatorial locomotion in rabbits
Source: PLoS Genet. 2021 Mar 25;17(3):e1009429. doi: 10.1371/journal.pgen.1009429 (PMC7993613; doi:10.1371/journal.pgen.1009429)
Supplement: S1 Table — (PDF) [file pgen.1009429.s002.pdf]

S1 Table. Whole genome resequencing and read mapping statistics

Pool	Number of individuals pooled	Number of reads	Percentage of reads mapping[a]	Percentage of reads MQ >=20[b]	Mean depth of coverage[c]
sauteur	12	884,371,195	98.7% (94.3%)	89.2%	37.6
wild-type	40	867,377,377	98.8% (94.4%)	94.0%	36.5

[a]Percentage of properly paired reads is given in parentheses
[b]Reads with a Phred score mapping quality (MQ) equal or higher than 20
[c]Including positions with zero reads mapping
