# Supplementary material for: A loss-of-function mutation in RORB disrupts saltatorial locomotion in rabbits
Source: PLoS Genet. 2021 Mar 25;17(3):e1009429. doi: 10.1371/journal.pgen.1009429 (PMC7993613; doi:10.1371/journal.pgen.1009429)
Supplement: S2 Table — (PDF) [file pgen.1009429.s003.pdf]

S2 Table. List of genes within the candidate region (chromosome1:59,560,684-64,953,774 bp).

Gene name/Ensembl ID	Gene description	Position (strand)
ANXA1	annexin A1	chr1:59691444-59711988 (+)
RPS27	40S ribosomal protein S27	chr1:60254357-60254611 (-)
RF00001	rRNA	chr1: 60383945-60384042 (-)
RPS14	40S ribosomal protein S14	chr1:60521557-60522084 (-)
RORB	RAR related orphan receptor B	chr1:60906485-61123665 (+)
TRPM6	transient receptor potential cation channel subfamily M member 6	chr1:61168958-61242200 (-)
C9orf40	chromosome 1 open reading frame, human C9orf40	chr1:61368482-61375500 (-)
CARNMT1	carnosine N-methyltransferase 1	chr1:61398007-61449350 (-)
NMRK1	nicotinamide riboside kinase 1	chr1:61476176-61495324 (-)
OSTF1	osteoclast stimulating factor 1	chr1:61502198-61562877 (+)
PCSK5	proprotein convertase subtilisin/kexin type 5	chr1:62290512-62721376 (+)
RFK	riboflavin kinase	chr1:62749021-62763820 (-)
GCNT1	glucosaminyl (N-acetyl) transferase 1	chr1:62871871-62873157 (+)
PRUNE2	prune homolog 2 with BCH domain	chr1:63004202-63320005 (-)
FOXB2	forkhead box B2	chr1:63425596-63427427 (+)
RF00026	snRNA	chr1: 63535542-63535648 (-)
VPS13A	vacuolar protein sorting 13 homolog A	chr1:63590621-63836968 (+)
GNA14	G protein subunit alpha 14	chr1:63848020-64070836 (-)
GNAQ	guanine nucleotide-binding protein G(q) subunit alpha	chr1:64143577-64340449 (-)
CEP78	centrosomal protein 78	chr1:64623120-64675004 (+)
PSAT1	phosphoserine aminotransferase 1	chr1:64697635-64728069 (+)
