# Supplementary material for: A loss-of-function mutation in RORB disrupts saltatorial locomotion in rabbits
Source: PLoS Genet. 2021 Mar 25;17(3):e1009429. doi: 10.1371/journal.pgen.1009429 (PMC7993613; doi:10.1371/journal.pgen.1009429)
Supplement: S3 Table — Each cell indicates the percentage of reads of each isoform (read counts are shown in parenthesis). (PDF) [file pgen.1009429.s004.pdf]

S3 Table. Relative abundance of the four most common *RORB* isoforms in rabbit retina and spinal cord quantified through Nanopore sequencing of amplicons obtained from cDNA. Each cell indicates the percentage of reads of each isoform (read counts are shown in parenthesis).

	**Retina**			**Spinal cord**		
	**wild type (+/+)**	**heterozygote (+/s^{am})**	**sauteur (s^{am}/s^{am})**	**wild type (+/+)**	**heterozygote (+/s^{am})**	***sauteur*** **(s^{am}/s^{am})**
Isoform 1	100.0% (4162)	87.1% (525)	12.6% (127)	99.6% (6797)	57.9% (232)	12.4% (87)
Isoform 2	0.0% (0)	6.1% (37)	35.8% (361)	0.4% (26)	10.5% (42)	33.3% (233)
Isoform 3	0.0% (0)	3.3% (20)	37.2% (375)	0.0% (0)	31.4% (126)	53.9% (377)
Isoform 4	0.0% (0)	3.5% (21)	14.4% (145)	0.0% (0)	0.2% (1)	0.4% (3)
