# Supplementary material for: A loss-of-function mutation in RORB disrupts saltatorial locomotion in rabbits
Source: PLoS Genet. 2021 Mar 25;17(3):e1009429. doi: 10.1371/journal.pgen.1009429 (PMC7993613; doi:10.1371/journal.pgen.1009429)
Supplement: S4 Table — (PDF) [file pgen.1009429.s005.pdf]

S4 Table. List of primers used in this study

	Primer 1	Primer 2	Position	Notes
**Genotyping**				
RORB	TTCTGATGACCTAGTGAATGAAGC	CCAAAAATATAAAAACTTTGCTAAGGA	Chr1:61103393-61103643	
**Isoform quantification**				
RORB	(TCGTCGGCAGCGTCAGATGTGTATAAGAGACAG) CAGTGTGCCATCCAGATCAC	(GTCTCGTGGGCTCGGAGATGTGTATAAGAGACAG) TGGCACAGTCAGGGTTAAAGA	Chr1:61093796-61123651	Sequence in parenthesis – tail sequence for adapters
**qPCR**				
RORB_exons7-8	CAGTGTGCCATCCAGATCAC	TGAACATCTGCATTCCTCCA	Chr1:61093796-61100666	Spanning exons 7-8
RORB_exons10-11	CGAGCTTGGCTGATAGAACC	CAAACTGCAGTGATGGTTGG	Chr1:61108344- 61123544	Spanning exons 10-11
GAPDH	GCCCAGAACATCATCCCTGC	CGTATTTGGCAGCTTTCTCC	Chr9:10338374-(*)	Housekeeping gene; *reverse primer designed based on Diribarne *et al.*, 2012

*Diribarne, M., Mata, X., Rivière, J., Bouet, S., Vaiman, A., Chapuis, J., ... & Allain, D. (2012). LIPH expression in skin and hair follicles of normal coat and Rex rabbits. PloS one, 7(1), e30073.
