# Supplementary material for: A loss-of-function mutation in RORB disrupts saltatorial locomotion in rabbits
Source: PLoS Genet. 2021 Mar 25;17(3):e1009429. doi: 10.1371/journal.pgen.1009429 (PMC7993613; doi:10.1371/journal.pgen.1009429)
Supplement: Attachment — Submitted filename: Response to the reviewers_for_submission.docx [file pgen.1009429.s007.docx]

Dear Professors Gregory Copenhaver and Gregory Barsh:

In response to the helpful comments and suggestions from the reviewers, we have revised our manuscript. We have addressed the comments of the two reviewers by performing new analysis and making the necessary corrections and clarifications. We interleave our responses with the comments below. We hope that with these revisions the paper is now ready for publication in PLoS Genetics. Thank you for your consideration.

**Reviewer #1 comments:**

**The manuscript describes a very interesting study in a very peculiar rabbit line/strain, the sauteur d'Alfort rabbit, that exibits a defective locomotion behaviour. The authors provided several evidences supporting the causative role of a splice site mutation in the RORB gene opening new lines of research toward the complete characterization and role of RORB in gait. The authors identified the putative causative mutation by whole genome resequencing by applying a bulked segregant analysis based on an F2 family and reported gene expression and histochemical evidences.**

**I have just a couple of suggestions:**

**1) The title could be misleading - I would suggest to readdress it to what the work was able to demonstrate - that means that a mutation affecting the RORB gene expression determines an anomalous locomotion.**

We have followed the reviewer’s recommendation and modified the title.

**2) Sauter d'Alfort rabbits are traditionally considered as a strain or as a line and not a breed. This might be corrected in the introduction and in the abstract.**

The term “breed” has been replaced by “strain” throughout the manuscript.

**Reviewer #2 comments:**

**In this study by Carneiro et al. use a domestic rabbit breed, the sauteur d’Alfort, which has an abnormal bipedal locomotor gait. Whole genome sequencing analysis identified a mutation at a single locus containing RORB, which could explain this abnormal gait. I am not in the position to critically evaluate the validity of the genetic analysis and sequencing and therefore I will focus my critique on the IHC data and their interpretation. This study is of interest and potentially suitable for publication in PLOS Genetics, there are however some weak points that need to be addressed in a revised manuscript.**

**1) Figure 4: The changes in the number of neurons is mostly limited to the dorsal part of the spinal cord. Most of the neurons involved in the generation and coordination of locomotion are located in the ventral part of the spinal cord. Is there any explanation as to whether this is the case? Some discussion of these observations is necessary.**

It is true that most of the neurons involved in the coordination of locomotion are localized in the ventral part of the spinal cord. However, it was shown that interneurons located in the deep dorsal horn and receiving inputs from low threshold mechanosensory primary afferents play a role in the regulation of fine motor control (Bourane et al. 2015, Paixao et al. 2019, Abraira et al. 2017). Following the reviewer’s suggestion, a paragraph has now been added to the Discussion section about the contribution of these dorsal horn interneurons in the control of motor functions (Page 13 Line 319 to Page 14 Line 344).

**2) The results of Figure 5 are not convincing and need to be quantified appropriately. High magnification pictures with quantification should be provided for all panels to allow the reader to assess these results. I suggest quantifying the number of neurons along the dorso-ventral axis.**

The reviewer raises valid points. We addressed this in two ways. First, dorso-ventral quantifications of the number of neurons have been performed for the four immunohistochemistry experiments. Graphs and statistics describing these results were added to Figure 5. Second, we have also added magnified pictures of the dorsal horn for the immunohistochemistry with Calbindin and SatB1/2.

**3) DMRT3 expression and distribution was only affected in 3 of 6 rabbits while the remaining 3 were not affected. This raises doubts about the validity of these results and whether the observed changes in DMRT3 expression contributes to the phenotype. The conclusion that the differentiation of DMRT3 neurons is affected in these animals is not fully supported.**

After having performed the quantification of the number of Dmrt3 neurons both for the three *sauteur* rabbits with misplaced Dmrt3 neurons above the central canal and for the three remaining rabbits, we observed that for the latter one, although the Dmrt3 neurons are not misplaced, their number is larger than in control animals. This suggests that the differentiation of the Dmrt3 neurons is affected in all *sauteur* rabbits albeit not with the same degree of severity, which may also explain the difference of severity observed for the locomotor phenotype. This is now explicitly mentioned in the Results (Page 11 Line 267-272) and Discussion sections (Page 15 Line 355-357).
